# Corrected QT Interval Is Associated With Stroke but Not Coronary Heart Disease: Insights From a General Chinese Population

**DOI:** 10.3389/fcvm.2021.605774

**Published:** 2021-07-21

**Authors:** Xiaofan Guo, Zhao Li, Ying Zhou, Shasha Yu, Hongmei Yang, Guozhe Sun, Liqiang Zheng, Byron K. Lee, Mark J. Pletcher, Yingxian Sun

**Affiliations:** ^1^Department of Cardiology, The First Hospital of China Medical University, Shenyang, China; ^2^Department of Clinical Epidemiology, Library, Shengjing Hospital of China Medical University, Shenyang, China; ^3^Division of Cardiology, Department of Medicine, University of California, San Francisco, San Francisco, CA, United States; ^4^Department of Epidemiology and Biostatistics, University of California, San Francisco, San Francisco, CA, United States

**Keywords:** QTc interval, electrocardiogram, cardiovascular disease, stroke, coronary heart disease

## Abstract

**Background:** Prolonged heart rate-corrected QT (QTc) interval has been associated with incident cardiovascular diseases (CVD) in general Western populations. However, this association is unclear in Asian population. We aim to estimate the association between QTc interval and incident CVD in a general Chinese population.

**Methods:** We analyzed 8,867 participants age ≥35 years and free of CVD at baseline in the Northeast China Rural Cardiovascular Health Study. A resting 12-lead electrocardiogram was performed on all participants, and QTc interval computed using the Framingham formula. Cox proportional hazards models were used to calculate hazard ratios (HRs) with 95% confidence intervals (CIs) for associations between QTc interval and incident stroke, coronary heart disease, and combined CVD events.

**Results:** Over a median follow-up of 4.66 years, a total of 439 CVD events occurred (298 stroke cases and 152 CHD cases). After full adjustment, prolonged QTc defined by a sex-specific cutoff was associated with increased risk of developing stroke (HR: 1.82, 95% CI 1.20–2.75, *P* = 0.004) and combined CVD (HR: 1.52, 95% CI 1.05–2.19, *P* = 0.026). Spline analyses demonstrated no clear thresholds; when modeled as a linear relationship, each 10 ms increase of QTc interval was associated with an HR of 1.12 (95% CI 1.06–1.19, *P* < 0.001) for stroke and an HR of 1.10 (95% CI 1.05–1.15, *P* < 0.001) for combined CVD. Baseline QTc interval was not associated with incident CHD with either modeling strategy.

**Conclusions:** Baseline QTc interval is associated with incident stroke and CVD in adults without prior CVD from a general Chinese population.

## Introduction

In China, cardiovascular disease (CVD) is still the leading cause of death, although the epidemiological features are changing over time (1). The number of CVD deaths per year increased from 2.51 million in 1990 to 3.97 million in 2016, with coronary heart disease, ischemic stroke, and hemorrhagic stroke the top three causes (2). It is estimated that ~290 million patients presently suffer from CVD in China, most prominently in rural areas where CVD mortality has exceeded the rate in urban centers (3). Given the continuously rising health burden, CVD remains the most crucial public health issue in China.

Several studies have associated heart rate-corrected QT (QTc) interval with stroke (4, 5), with coronary heart disease (6), and with CVD on a general population level (7–10), suggesting the prognostic value of QTc interval for primary prevention of CVD. However, the evidence is inconsistent and largely from Western populations (4, 6, 9, 10). It has been suggested that there are race-specific differences in QTc interval and its association with CVD risk (6, 11, 12), but evidence from Asian populations is scarce.

The Northeast China Rural Cardiovascular Health Study (NCRCHS) provides a unique opportunity to address this knowledge gap. NCRCHS collected baseline risk CVD risk factors, electrocardiograms, and CVD events over time in a large general Chinese population. It is comparable to recent large cohorts conducted in Western populations in study design, sample size, method used for QTc interval calculation and the variables adjusted. We thus used NCRCHS to estimate the association between QTc interval and incident CVD.

## Methods

### Study Population

NCRCHS is a community-based prospective cohort study carried out in rural areas of Northeast China. The design and inclusion criteria of the study have been described previously (13, 14). In brief, a total of 11,956 participants aged ≥35 years were recruited from Dawa, Zhangwu, and Liaoyang counties in Liaoning province between 2012 and 2013, using a multi-stage, randomly stratified cluster-sampling scheme. Detailed information was collected at baseline for each participant. In 2015 and 2017, participants were invited to attend a follow-up study. Of the 11,956 subjects, 10,700 participants consented and were qualified for our follow-up study. A total of 10,349 participants completed at least one follow-up visit. The study was approved by the Ethics Committee of China Medical University (Shenyang, China), and written informed consent was obtained from all participants.

In the current analyses, we excluded participants with CVD at baseline (*n* = 821), participants with an electrocardiogram (ECG) unsuitable for QT interval measurement (*n* = 292), including QRS duration ≥120 ms, bundle branch blocks, second and third degree AV-blocks, pacemaker or implantable cardioverter-defibrillator implantation, multiple premature ventricular complexes, multiple premature atrial complexes, WPW syndrome and poor quality ECG data, and participants with missing or abnormal data (*n* = 369). Data were therefore available for 8,867 qualifying participants.

### Study Variables

At baseline, detailed information on demographic characteristics, dietary and lifestyle factors, and medical history were obtained by interview with a standardized questionnaire. Smoking status was defined as current use. History of stroke and coronary heart disease (CHD) at baseline was defined as self-reported and confirmed by medical records. Current use of aspirin, antihypertensive medications, statins, warfarin, and QT-prolonging medications was self-reported. We defined QT-prolonging medications according to previous literature (9, 15). Weight and height were measured with participants in lightweight clothing and without shoes. Waist circumference (WC) was measured at the umbilicus using a non-elastic tape. Body mass index (BMI) was computed as weight in kilograms divided by the square of height in meters. Obesity was defined as BMI ≥ 28 kg/m^2^ (16). Blood pressure was assessed three times with participants seated after at least 5 min of rest using a standardized automatic electronic sphygmomanometer (HEM-907; Omron, Tokyo, Japan). Hypertension was defined as systolic blood pressure (SBP) ≥ 140 mm Hg and/or diastolic blood pressure (DBP) ≥ 90 mm Hg, and/or use of antihypertensive medications (17). Fasting blood samples were collected in the morning from participants who had fasted at least 12 h. Fasting plasma glucose (FPG), total cholesterol (TC), low-density lipoprotein cholesterol (LDL-C), high-density lipoprotein cholesterol (HDL-C), triglyceride (TG), serum creatinine, and other routine blood biochemical indexes were analyzed enzymatically. Diabetes mellitus was defined as FPG ≥ 7 mmol/L (126 mg/dl) and/or being on medication for diabetes (18). Metabolic syndrome (MS) was defined by meeting three or more of the following component risk factors: (1) WC ≥ 90 cm for males and ≥80 cm for females, (2) BP ≥ 130/85 mm Hg or current use of antihypertensive drugs, (3) serum glucose level ≥ 5.6 mmol/L or current use of antihyperglycemic agents, (4) serum TGs ≥ 1.7 mmol/L, and (5) HDL-C <1.0 mmol/L for males and <1.3 mmol/L for females, according to the Adult Treatment Panel-III (ATP-III) modified criteria (19). Estimated glomerular filtration rate (eGFR) was calculated using the Chronic Kidney Disease Epidemiology Collaboration (CKD-EPI) formula (20).

### Electrocardiography

Twelve-lead ECGs (resting, 10 s) were performed on all participants by well-trained cardiologists at baseline using a MAC 5500 (GE Healthcare; Little Chalfont, Buckinghamshire, UK) at 10 mm/mV calibration and speed of 25 mm/s, and analyzed automatically by the MUSE Cardiology Information System, version 7.0.0 (GE Healthcare). ECG parameters including QT interval were measured automatically with QT interval corrected for heart rate using the Framingham formula (21), a linear-scaled correction formula. Prolonged QTc was defined as QTc ≥ 460 ms for females and ≥450 ms for males (22). Atrial fibrillation (AF) was diagnosed based on previous history diagnosed by a physician or ECG findings. Electrocardiographic left ventricular hypertrophy (LVH) was defined per Sokolow–Lyon criteria (23).

### Adjudication of Endpoints

Median follow-up was 4.66 years. In the present study, an incident CVD event was defined as a composite of new onset Stroke or CHD during the follow-up period. The specific incidences of stroke and CHD were also determined. For all participants reporting possible diagnoses or death, all available clinical information was collected including medical records and death certificates. All materials were independently reviewed and adjudicated by the end-point assessment committee. Stroke was defined per WHO Multinational Monitoring of Trends and Determinants in Cardiovascular Disease (MONICA) criteria (24, 25) as rapidly developing signs of focal or global disturbance of cerebral function, lasting more than 24 h (unless interrupted by surgery or death), with no apparent non-vascular cause. Transient ischemic attack and chronic cerebral vascular disease were excluded. CHD was defined as a diagnosis of hospitalized angina, hospitalized myocardial infarction, CHD death, or any revascularization procedure (26).

### Statistical Analysis

Descriptive statistics were calculated for all variables, including continuous variables (reported as mean values and standard deviations) and categorical variables (reported as numbers and percentages). Differences among categories were evaluated using *t*-test, non-parameter test, or χ2-test as appropriate. Kaplan-Meier methods were used to estimate the cumulative incidence of stroke, CHD, and combined CVD in participants with and without prolonged QTc interval, and log-rank testing used to compare differences in estimates. Cox proportional hazards models were used to estimate adjusted associations between QTc interval and the risk of CHD, stroke, and combined CVD incidence with hazard ratios (HRs) and 95% confidence intervals (CIs) calculated. We constructed four models with sequential adjustment. Model 1 is adjusted for age, sex and ethnicity. Model 2 is further adjusted for current smoking, family history of coronary heart disease, BMI, SBP, antihypertensive medications, FPG, LDL-C, TG, HDL-C, TC, and eGFR. Model 3 is adjusted for the previous factors as well as QT-prolonging medications, statin use, warfarin use, and aspirin use. And Model 4 is adjusted for the full range of Model 3 covariates plus AF and ECG-LVH. We also modeled QTc with restricted cubic splines to illustrate the linearity of the dose-response relationship between QTc interval and each endpoint. In spline analyses, we used the 50th percentile of the QTc distribution as the reference value. In addition, we evaluated the association between QTc interval and cardiovascular events stratified by age, sex, smoking status, hypertension, obesity, diabetes, and MS, and calculated nominal interaction *p*-values with and without adjustment for multiple comparisons via Bonferroni correction. All statistical analyses were performed using Stata version 14.0 and SPSS version 17.0 software, and *P* < 0.05 were considered statistically significant.

## Results

The characteristics of the study participants stratified by prolonged QTc interval are shown in Table 1. The average QTc duration was 417.9 ± 19.9 ms. A total of 284 (3.2%) participants exhibited a prolonged QTc interval, and these participants were more likely to be of older age and have higher proportions of hypertension, diabetes and LVH.

**Table 1:** Baseline characteristics of the included participants by prolonged QTc interval.

	**Total**	**With prolonged QTc**	**Without prolonged QTc**	***P*-value**
	**(*n* = 8,867)**	**(*n* = 284)**	**(*n* = 85,83)**	
Age (year)	53 ± 10	58 ± 11	53 ± 10	<0.001
Male (%)	4,087 (46.1)	122 (43.0)	3,965 (46.2)	0.281
Ethnicity of Han (%)	8,344 (94.1)	266 (93.7)	8,078 (94.1)	0.749
Current smoking (%)	3,165 (35.7)	103 (36.3)	3,062 (35.7)	0.838
BMI (kg/m^2^)	24.8 ± 3.7	25.2 ± 4.8	24.8 ± 3.6	0.228
SBP (mmHg)	141.0 ± 22.9	154.9 ± 28.2	140.6 ± 22.5	<0.001
FPG (mmol/L)	5.8 ± 1.5	5.9 ± 1.9	5.8 ± 1.5	0.422
TC (mmol/L)	5.2 ± 1.1	5.3 ± 1.0	5.2 ± 1.1	0.293
TG (mmol/L)	1.6 ± 1.4	1.6 ± 1.2	1.6 ± 1.4	0.71
HDL-C (mmol/L)	1.4 ± 0.4	1.5 ± 0.5	1.4 ± 0.4	0.049
LDL-C (mmol/L)	2.9 ± 0.8	3.0 ± 0.8	2.9 ± 0.8	0.228
Estimated GFR (ml/min/1.73 m^2^)	94.3 ± 15.0	93.1 ± 15.7	94.4 ± 15.0	0.179
Family history of CHD (%)	1,222 (13.8)	41 (14.4)	1,181 (13.8)	0.745
Obesity (%)	1,526 (17.2)	52 (18.3)	1,474 (17.2)	0.618
Hypertension (%)	4,337 (48.9)	198 (69.7)	4,139 (48.2)	<0.001
Diabetes (%)	815 (9.2)	39 (13.7)	776 (9.0)	0.007
Metabolic syndrome (%)	3,230 (36.4)	115 (40.5)	3,115 (36.3)	0.148
Antihypertensive medication (%)	1,073 (12.1)	67 (23.6)	1,006 (11.7)	<0.001
QT prolonging medication (%)	162 (1.8)	7 (2.5)	155 (1.8)	0.415
Statin use (%)	7 (0.1)	0 (0.0)	7 (0.1)	NA
Warfarin use (%)	2 (0.0)	0 (0.0)	2 (0.0)	NA
Aspirin use (%)	68 (0.8)	3 (1.1)	65 (0.8)	0.481
Atrial fibrillation (%)	56 (0.6)	2 (0.7)	54 (0.6)	0.7
Left ventricular hypertrophy (%)	1,150 (13.0)	77 (27.1)	1,073 (12.5)	<0.001
Heart rate (beats/min)	72 ± 12	68 ± 11	72 ± 12	<0.001
QTc interval (ms)	417.9 ± 19.9	466.6 ± 13.4	416.3 ± 18.0	<0.001

*Data are expressed as the mean ± SD or as n (%)*.

*Prolonged QTc defined as 460 ms or longer in females and 450 ms or longer in males using Framingham formula*.

*BMI, body mass index; CHD, coronary heart disease; FPG, fasting plasma glucose; GFR, glomerular filtration rate; HDL-C, high-density lipoprotein cholesterol; LDL-C, low-density lipoprotein cholesterol; NA, not applicable; SBP, systolic blood pressure; TC, total cholesterol; TG, triglyceride*.

**Figure 1:**
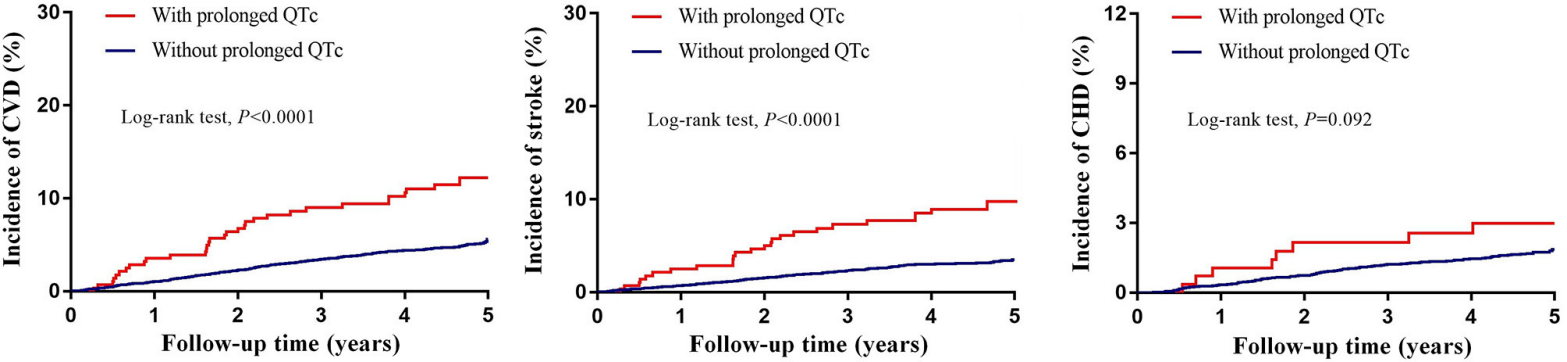
Unadjusted Kaplan–Meier curves for incident cardiovascular events stratified by prolonged QTc interval. CHD, coronary heart disease; CVD, cardiovascular disease. Prolonged QTc interval defined as 460 ms or longer in females and 450 ms or longer in males using Framingham formula.

During follow-up, a total of 439 CVD events were documented (298 stroke cases and 152 CHD cases). The Kaplan-Meier curves for each endpoint in participants with and without prolonged QTc interval are shown in Figure 1. Higher cumulative incidences of stroke and total CVD events (but only insignificantly higher incidence of CHD) were observed among all participants with prolonged QTc interval.

Table 2 presents the adjusted associations of baseline QTc interval with incident CVD events over the follow-up period. Prolonged QTc was associated with increased risk of developing stroke (HR: 1.82, 95% CI 1.20–2.75, *P* = 0.004) and combined CVD (HR: 1.52, 95% CI 1.05–2.19, *P* = 0.026) even after full adjustment for all covariates including AF and ECG-LVH (Models 1–4). Each 10 ms increase of QTc interval was associated with an HR of 1.12 (95% CI 1.06–1.19, *P* < 0.001) for stroke and an HR of 1.10 (95% CI 1.05–1.15, *P* < 0.001) for combined CVD after full adjustment. Baseline QTc interval was not associated with incident CHD either in dichotomous or continuous models.

**Table 2:** Multivariate-adjusted hazard ratios for combined cardiovascular disease, stroke, and coronary heart disease associated with QTc interval.

		**Model 1**		**Model 2**		**Model 3**		**Model 4**	
	**n/N**	**HR (95% CI)**	***P*-value**	**HR (95% CI)**	***P*-value**	**HR (95% CI)**	***P*-value**	**HR (95% CI)**	***P*-value**
**PROLONGED QTc**									
Combined CVD	439/8,867	1.76 (1.23–2.53)	0.002	1.55 (1.08–2.24)	0.018	1.56 (1.08–2.25)	0.017	1.52 (1.05–2.19)	0.026
Stroke	298/8,867	2.15 (1.43–3.22)	<0.001	1.85 (1.23–2.79)	0.003	1.87 (1.24–2.82)	0.003	1.82 (1.20–2.75)	0.004
CHD	152/8,867	1.29 (0.63–2.65)	0.481	1.16 (0.57–2.38)	0.686	1.15 (0.56–2.36)	0.704	1.10 (0.54–2.27)	0.793
**PER 10 MS INCREASE IN QTc**									
Combined CVD	439/8,867	1.14 (1.09–1.19)	<0.001	1.10 (1.05–1.15)	<0.001	1.10 (1.05–1.16)	<0.001	1.10 (1.05–1.15)	<0.001
Stroke	298/8,867	1.17 (1.11–1.24)	<0.001	1.13 (1.06–1.19)	<0.001	1.13 (1.07–1.19)	<0.001	1.12 (1.06–1.19)	<0.001
CHD	152/8,867	1.10 (1.01–1.19)	0.025	1.06 (0.97–1.15)	0.187	1.06 (0.97–1.15)	0.196	1.05 (0.97–1.15)	0.215

*Model 1: adjusted for age, sex and ethnicity*.

*Model 2: adjusted for model 1 + current smoking, family history of coronary heart disease, body mass index, systolic blood pressure, antihypertensive medications, fasting plasma glucose, total cholesterol, triglyceride, high-density lipoprotein cholesterol, low-density lipoprotein cholesterol and estimated glomerular filtration rate*.

*Model 3: adjusted for model 2 + QT prolonging medications, statin use, warfarin use and aspirin use*.

*Model 4: adjusted for model 3 + atrial fibrillation and left ventricular hypertrophy*.

*Prolonged QTc defined as 460 ms or longer in females and 450 ms or longer in males using Framingham formula. n means the number of events; N means the number of study population*.

*CHD, coronary heart disease; CI, confidence interval; CVD, cardiovascular disease; HR, hazard ratio*.

**Figure 2:**
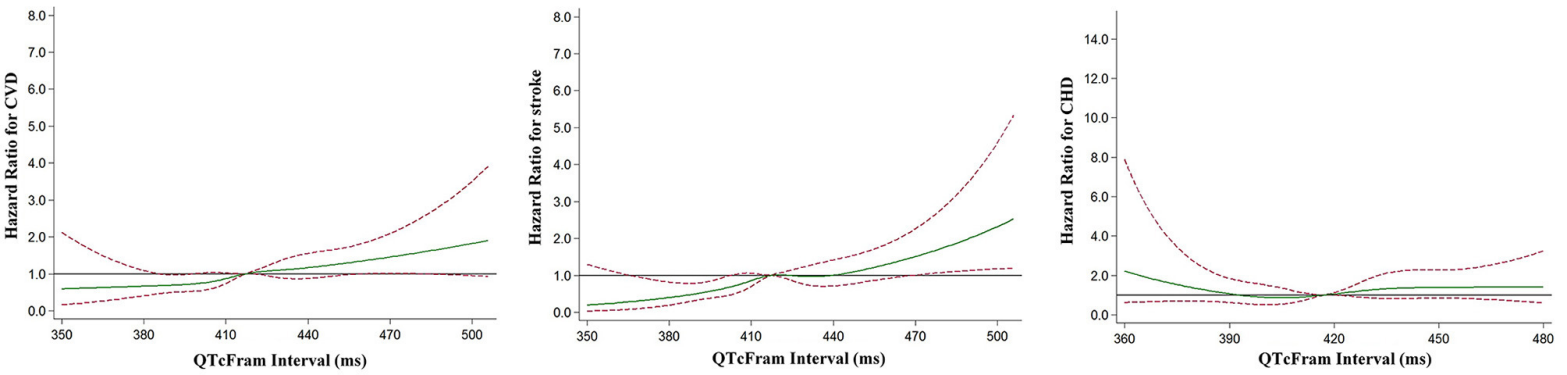
Multivariable-adjusted restricted cubic spline analysis for the hazard ratio of cardiovascular events as a function of the QTc interval. The green solid line indicates multivariable-adjusted hazard ratios for cardiovascular events, and the red dashed lines indicate the upper and lower 95% confidence intervals. The horizontal black line indicates a hazard ratio of 1. Adjustment factors are as Model 4 in Table 2. CHD, coronary heart disease; CVD, cardiovascular disease.

We then used restricted cubic splines to provide a detailed analysis of the dose-response relationship of the QTc with incident CVDs. We found no clear evidence of a threshold effect in adjusted associations modeled via restricted cubic splines. The risk of stroke and combined CVD progressively increased with increasing QTc intervals (Figure 2).

**Figure 3:**
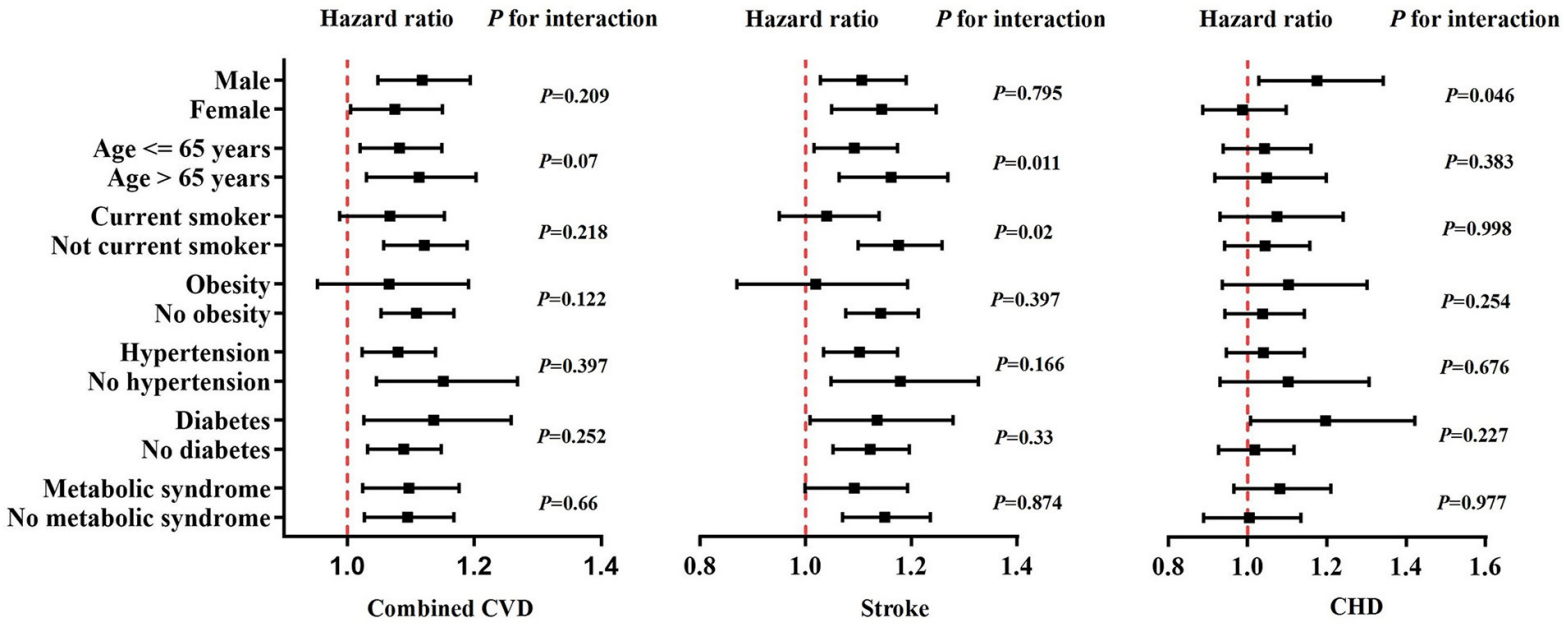
Multivariate-adjusted hazard ratios for cardiovascular events associated with each 10 ms increase of QTc interval within subgroups of the study participants. Black squares in the graph represent the hazard ratio and lines represent the 95% confidence interval. The vertical red line indicates a hazard ratio of 1. Adjustment factors are as Model 4 in Table 2. CHD, coronary heart disease; CVD, cardiovascular disease.

Figure 3 shows the multivariable-adjusted HRs, per 10 ms increase of QTc interval, across subgroups. We found borderline nominal interactions with stronger associations for stroke in older persons (>65 years, interaction *P* = 0.011) and non-smokers (*P* = 0.02), and for CHD in males (*P* = 0.046). These interactions were not significant with adjustment for multiple comparisons.

## Discussion

**Figure 4:**
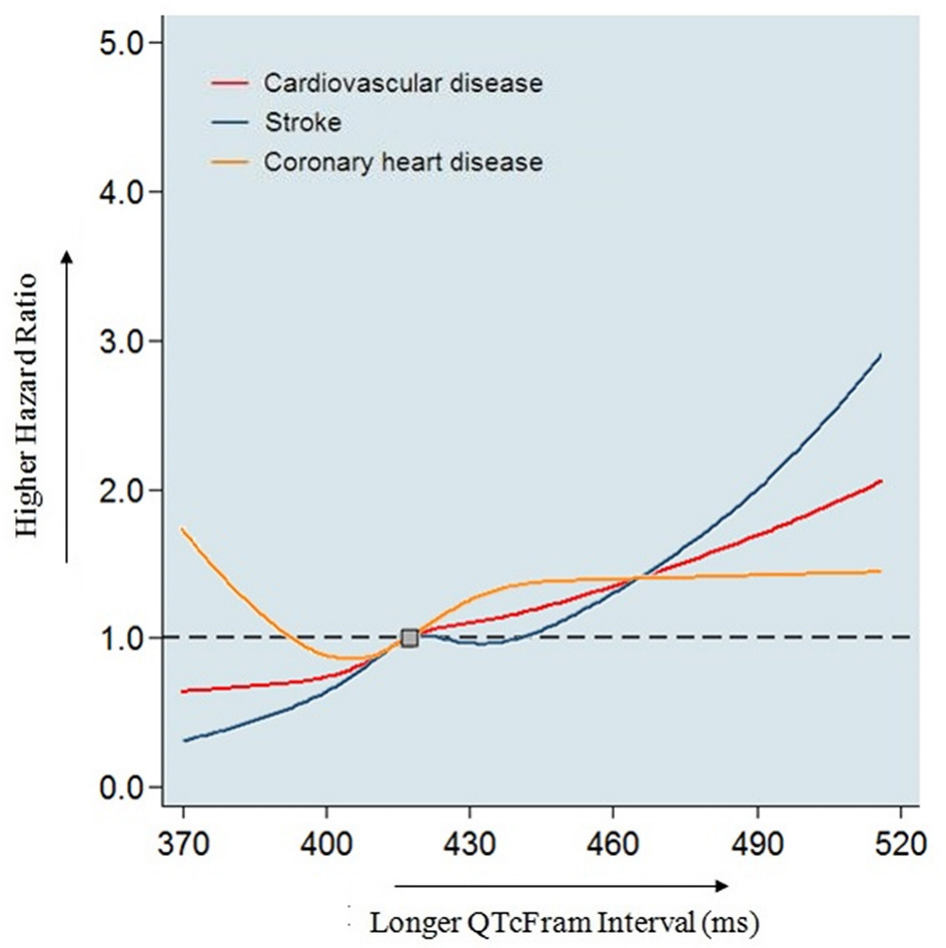
Association of baseline QTc interval with coronary heart disease, stroke, and combined cardiovascular disease in 8,867 Chinese participants. Models adjusted for age, sex, ethnicity, current smoking, family history of coronary heart disease, body mass index, systolic blood pressure, antihypertensive medications, fasting plasma glucose, total cholesterol, triglyceride, high-density lipoprotein cholesterol, low-density lipoprotein cholesterol and estimated glomerular filtration rate, QT prolonging medications, statin use, warfarin use and aspirin use, atrial fibrillation and left ventricular hypertrophy. In this study, we found prolonged QTc interval was associated with incident stroke and combined cardiovascular disease, but not coronary heart disease in adults without prior cardiovascular disease in a general Chinese population.

In this large general Chinese population, we found that baseline QTc interval was significantly associated with a higher risk of incident stroke and CVD. This association was independent of established CVD risk factors, atrial fibrillation, left ventricular hypertrophy, and estimated glomerular filtration rate (Figure 4). These findings add further evidence regarding the significance of QTc interval prolongation in a specific population (China) with a distinct pattern of CVD.

Prolonged QTc interval reflects prolonged ventricular repolarization, which can lead to ventricular fibrillation and sudden death. Very recently, QTc interval has been proposed as an emerging vital sign (27), but its predictive value in the general Chinese population remains unknown. Although there is considerable evidence for the link between QTc interval and CVD events in the literature, the results are inconsistent. For example, a review of seven prospective cohort studies found that prolonged QTc interval was not associated with increased risks of total or CVD mortality and sudden death in patients without established CVD (8). However, a meta-analysis found consistent associations between prolonged QT interval and increased risk of these outcomes, although retrospective and nested-case cohort studies were included (28). Two studies conducted in the general Japanese population found positive links between QTc interval and CVD risk, but both adopted Bazett's formula (29, 30) and this method is known to potentially overestimate the number of patients with prolonged QTc interval and its association with outcomes (31, 32). Therefore, in the present study we used the Framingham formula, a linear regression method, to adjust the QT interval for heart rate, which has been recommended in recent guidelines (22).

Studies on the relationship between QTc interval and CVD risk have continued to emerge in recent years, and the dust has not yet settled. A MESA study found that QTc interval was associated with incident cardiovascular events in adults age 45 or older who were free of CVD at baseline (10). Nielsen et al. observed pronounced associations between QTc interval and risk of CVD in the elderly and in those with prior CVD from a large primary care population (9). In the REGARDS study, Soliman et al. reported a strong association between QTc interval and incident stroke after adjustment for traditional stroke risk factors among 27,411 participants (4). Comparable to these studies conducted in Western populations, our study confirmed the impact of prolonged QTc interval on CVD outcomes in an Asian population. Most recently, it was found that ECG-LVH and prolonged QTc interval were both independent markers of poor prognosis, and the combination was associated with higher risk (5, 33, 34). We therefore additionally adjusted for ECG-LVH in the present study, which is different from most earlier studies of the QTc interval. Studies demonstrated that QTc interval was associated with intima-media thickness of carotid artery (35, 36), which was an established marker for subclinical atherosclerosis. This may in part explain the higher CVD risk associated with prolonged QTc interval. Studies aimed to further clarify the underling mechanisms are needed.

Previous studies showed race-specific differences in the QT interval and its association with CVD outcomes (6, 11), including prominent race differences in QTc intervals between Whites and Asians (12). Seyerle et al. found the presence of considerable heterogeneity in multiple genetic loci that influence QT interval among racial or ethnic groups, which potentially contributes to these race-specific differences (37). Up to now, the predictive value of QTc interval for future CVD events in Asians has been unclear, highlighting the necessity of our present study conducted in a large general Chinese population.

We and other colleagues previously found that chronic kidney disease was associated with prolonged QTc interval (38–40). Similar with ECG-LVH, the combination of chronic kidney disease and QTc prolongation resulted in a higher risk of mortality compared with either condition alone (39). As such, we also adjusted for eGFR in the regression model of the present study, and still found a positive relationship between QTc interval and CVD outcomes.

Our study has several limitations. First, we evaluated the association of only a single automated measurement of QTc interval at baseline with incident CVD events. However, previous studies used the same measurement and found it feasible. Second, data on use of QT-prolonging and other medications were self-reported. In the studied rural population, the use of these specific medications is extremely low due to lack of health consciousness and poor economic conditions, and these medications are rare enough that they would likely be remembered accurately, which should minimize the effects on results. Finally, we focus on a Chinese population, and thus our result may not be generalizable to all Asians.

In conclusion, we observed an increased risk of incident stroke and CVD with QTc interval at baseline, providing evidence from a large general Chinese population for the first time. The effect of QTc prolongation on CVD events differed across subgroups. Our study suggests that QTc measurement has important predictive value and could potentially serve as a vital sign in Chinese adults.

## Data Availability Statement

The raw data supporting the conclusions of this article is currently not available. Please direct any queries to Xiaofan Guo, guoxiaofan1986@hotmail.com.

## Ethics Statement

The studies involving human participants were reviewed and approved by the Ethics Committee of China Medical University (Shenyang, China). The patients/participants provided their written informed consent to participate in this study.

## Author Contributions

ZL and YS contributed to the conception or design of the work. XG, YZ, SY, HY, GS, and LZ contributed to the acquisition, analysis, or interpretation of data for the work. XG drafted the manuscript. BL and MP critically revised the manuscript. All gave final approval to the manuscript.

## Conflict of Interest

The authors declare that the research was conducted in the absence of any commercial or financial relationships that could be construed as a potential conflict of interest.
